# Design of Acquisition Schemes and Setup Geometry for Anisotropic X-ray Dark-Field Tomography (AXDT)

**DOI:** 10.1038/s41598-017-03329-0

**Published:** 2017-06-09

**Authors:** Y. Sharma, F. Schaff, M. Wieczorek, F. Pfeiffer, T. Lasser

**Affiliations:** 1 Chair of Biomedical Physics, Department of Physics and School of BioEngineering, Technical University of Munich, 85748 Garching, Germany; 2 Computer Aided Medical Procedures, Technical University of Munich, 85748 Garching, Germany; 3 Department of Diagnostic and Interventional Radiology, Klinikum rechts der Isar, Technical University of Munich, 81675 München, Germany; 4 Institute for Advanced Study, Technical University of Munich, 85748 Garching, Germany

## Abstract

Anisotropic X-ray Dark-field Tomography (AXDT) is a new imaging technique for reconstructing the three-dimensional scattering profile within a sample using the dark-field signal measured in an X-ray grating interferometry setup. As in any tomographic measurement, the acquisition geometry plays a key role in the accurate reconstruction of the scattering information. More- over, the anisotropic nature of the dark-field signal poses additional challenges for designing the acquisition protocols. In this work, we present an efficient approach to measure scattering orientations spread over the unit sphere and prove its efficacy using the knowledge from conventional tomography. In addition, we conclude (using analytical and experimental results) that placing the gratings such that the grating bars make an angle of 45 degrees with respect to the vertical direction is the optimal setup configuration for AXDT.

## Introduction

X-ray imaging using a grating interferometer has gained much attention in recent years^1–3^. By analyzing the interference pattern created by a phase grating at specific downstream positions, we obtain three signals namely attenuation, phase contrast and dark-field. While the attenuation and phase-contrast signals originate from the attenuation and phase shift of the incident X-ray beam caused by the sample, the dark-field signal originates from the small-angle scattering caused by micron and sub-micron sized structures^4–8^. The dark-field signal is anisotropic^9–11^ which means that the measured signal depends on the relative orientation of the sample with respect to the propagation direction of X-rays and the orientation of the grating bars.

The anisotropic property of the dark-field signal can be utilized to obtain two-dimensional orientation of microstructures in the imaging plane by rotating the sample around the beam propagation direction in a technique known as X-ray Vector Radiography (XVR)^12, 13^. A single-shot variant of XVR was recently presented by Kagias *et al*.^14^ where a specialized grating was used to obtain the two-dimensional orientations without any sample rotation. Since XVR can only resolve orientations in a plane orthogonal to the direction of beam propagation, it is best suited for investigating relatively flat samples. Revol *et al*.^15^ presented a method to calculate the strength of scattering along certain a priori known orientations in different layers of a multi-layered composite material. Bayer *et al*.^9^ presented a three-dimensional extension of XVR, however, they were able to reconstruct only the projection of orientations onto a plane.

Malecki *et al*.^16^ presented the first fully three-dimensional directional dark-field imaging method and termed it “X-ray Tensor Tomography” (XTT). XTT, as the name suggests, approximates the scattering profile in every volume element by a symmetric rank-2 tensor. Vogel *et al*.^17^ presented several algorithmic improvements for the fast and efficient reconstruction of these tensors in three dimensions. The tensor approximation, however, is insufficient for recovering multiple scattering orientations in a single volume element which is especially relevant since the structures probed with this technique are typically smaller than the spatial resolution. To overcome this limitation, Wieczorek *et al*.^18^ presented a technique termed “Anisotropic X-ray Dark-field Tomography” (AXDT) which reconstructs the scattering profile as a spherical function represented using spherical harmonics. This method not only provides a more accurate and robust representation of the three-dimensional scattering profile but also allows for the extraction of multiple scattering orientations in a single volume element. Directional dark-field imaging in two and three dimensions, such as XVR or XTT, has been found useful for resolving orientations in composite materials made of carbon and glass fibers as well as in biological specimens such as bones and teeth^19–21^.

**Figure 1:**
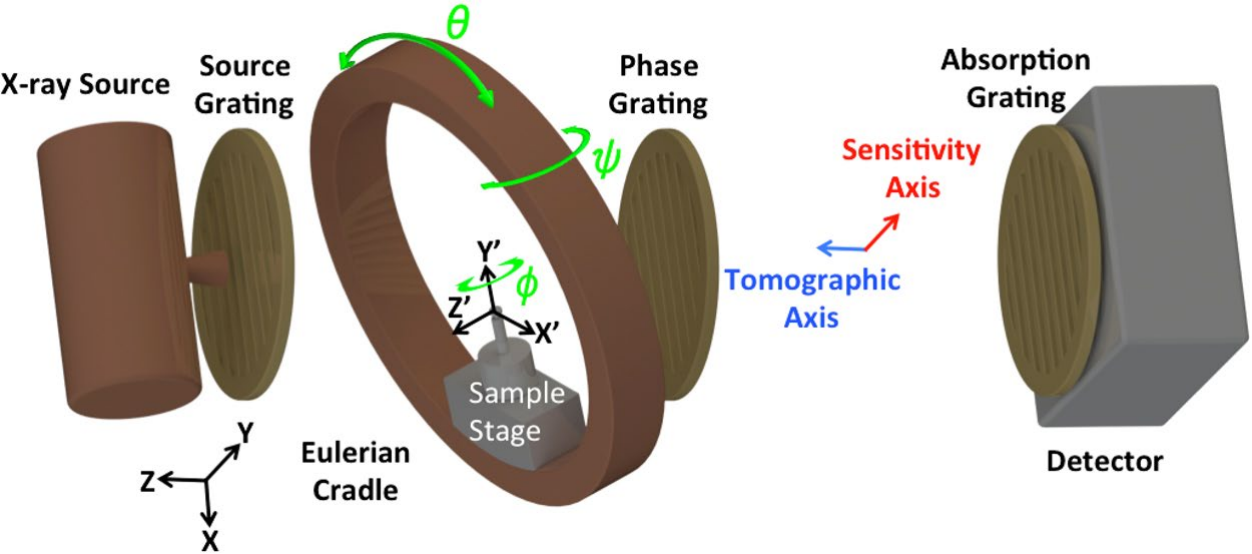
Schematic of a grating interferometer setup, as used in X-ray Tensor Tomography (XTT) and Anisotropic Dark-field Tomography (AXDT), showing a non-standard acquisition pose and the three axes of rotation. Figure by Sharma *et al*.^22^ is licensed under https://creativecommons.org/licenses/by/4.0/CC BY 4.0.

In order to reconstruct the three-dimensional scattering function accurately, the recorded anisotropic scattering information must be well distributed over all possible scattering orientations $q\in {{\mathbb{S}}}^{2}$ (we denote the border of the unit sphere in three dimensions as ${{\mathbb{S}}}^{2}=\{u\in {{\mathbb{R}}}^{3};|u|=1\}$). Malecki *et al*.^16^ presented a first idea of such a scheme utilizing the three rotation axes provided by an Eulerian cradle shown in Fig. 1. Sharma *et al*.^22^ presented a detailed analysis of similar schemes and introduced an analytical measure known as the “Coverage Metric” suitable for the relative comparison of XTT/AXDT acquisition schemes. These schemes were motivated mainly by convenience and speed of acquisition and even though they probe many scattering orientations as shown in our previous work^22^, all of these orientations are measured insufficiently.

In this work, we present an approach to acquire three-dimensional anisotropic dark-field data such that specific orientations on the unit sphere are probed completely. However, due to the physical limitations of the setup discussed in the next section, most of the orientations can only be probed up to a certain extent. We analyze the resulting acquisition schemes for different setup configurations and show that certain grating orientations are advantageous compared to others for measuring the most information within the physical limitations of the setup. In addition, we visualize the null space^23, 24^ of the proposed schemes to illustrate the correlation between the reconstruction and acquisition methods for AXDT. Finally, we corroborate the theoretical findings with experimental results for an industrially relevant fiber composite sample.

## Methods

This section is divided into three sub-sections. In the first sub-section, we present our novel concept of acquisition schemes and explain how to obtain such schemes mathematically. In the second sub-section, we present a short summary of Anisotropic X-ray Dark-field Tomography. Finally, we present a method for estimating one component of the null space of an AXDT acquisition scheme.

### Design of Acquisition Schemes

In conventional tomography, we acquire line integrals through a three-dimensional object onto a 2D detector and use analytic or iterative methods to reconstruct the 3D volume from several 2D images acquired at different poses of the sample. The pre-requisite for recovering the 3D spatial information from 2D projections is that the total measured signal in any projection is constant, that is, the measured quantity (such as X-ray attenuation coefficient) is invariant under rotation. This is, however, not true for the dark-field signal. Due to its anisotropic nature, the dark-field signal varies as the object is rotated around an axis. However, it is possible to define an axis of rotation such that a certain component of the scattering function (*q*) is invariant under rotation of the object around this particular axis^25^. We use this concept to design an acquisition trajectory that comprises of several poses for which a unique component of the dark-field signal remains invariant, thus allowing for a full tomographic reconstruction of this particular component. In the following, we explain how such an acquisition trajectory can be designed for any scattering orientation $q\in {{\mathbb{S}}}^{2}$.

We define the sensitivity vector $s(x)\in {{\mathbb{S}}}^{2}$ and the tomographic vector $t(x)\in {{\mathbb{S}}}^{2}$ for every acquisition pose *x* = (*ψ*, *θ*, *ϕ*), where the Euler angles *ψ* (rotation around *y*), *θ* (rotation around *z*′) and *ϕ* (rotation around *y*′) are shown in Fig. 1. We have:

1 $$\begin{array}{rcl}s(x) & = & R(x)\cdot S,\\ t(x) & = & R(x)\cdot T,\end{array}$$

where $R(x)\in {{\mathbb{S}}}^{3\times 3}$ is the Euler rotation matrix for the pose *x*, $S\in {{\mathbb{S}}}^{2}$ is the setup sensitivity, $T\in {{\mathbb{S}}}^{2}$ is the direction of beam propagation ([0, 0, 1]^*T*^ in the setup shown in Fig. 1) and · denotes standard matrix-vector multiplication. *S* is the direction in which the phase shift is measured by the grating interferometer setup; it is orthogonal to the grating bars in the plane of the gratings ([0, 1, 0]^*T*^ in the setup shown in Fig. 1). Evidently, the sensitivity vector denotes the scattering orientation that is probed at the acquisition pose *x* while the tomographic vector represents the direction along which the signal is integrated.

Let us define an acquisition scheme as:

2 $$A(\psi ,\theta ,N):=\{x=(\psi ,\theta ,\varphi );\varphi \in \{{0}^{\circ },\frac{{180}^{\circ }}{N},\ldots ,{180}^{\circ }-\frac{{180}^{\circ }}{N}\};N\in {\mathbb{N}}\}\mathrm{.}$$

**Figure 2:**
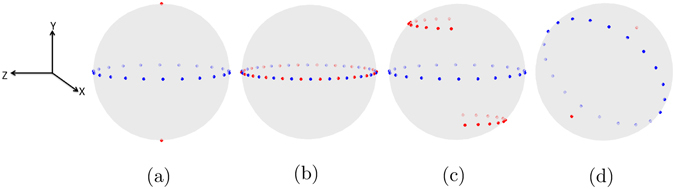
Spherical representation of acquisition schemes. Please note that in all these images, the sample can be imagined as being stationary at the center of the sphere while the setup rotates around it. Blue points represent the vectors ±*t*(*x*) and red points represent ±*s*(*x*) for all *x* ∈ *A*(0, 0, 11) with (**a**) horizontal grating alignment, (**b**) vertical grating alignment, and (**c**) diagonal grating alignment. (**d**) The vectors ± *t*(*y*) and ± *s*(*y*) for all *y* in an exemplary acquisition scheme *Y*(*q*, [0.7071, −0.7071, 0]^*T*^, 10) obtained using the method explained in Algorithm 1.

A conventional X-ray CT acquisition scheme in this notation can be expressed as *A*(0, 0, *N*). The points ±*t*(*x*) for *x* ∈ *A*(0, 0, 11) are shown as blue dots in Fig. 2(a). Such a circular measurement trajectory with sufficiently large value of *N* is desired for analytic reconstruction in X-ray CT. However, the sensitivity vector has no significance in X-ray CT since the measured quantity is invariant under rotation. In the case of dark-field signal, we also compute ±*s*(*x*) for *x* ∈ *A*(0, 0, 11) assuming horizontally (*S* = [0, 1, 0]^*T*^), vertically (*S* = [1, 0, 0]^*T*^) and diagonally (*S* = [0.7071, −0.7071, 0]^*T*^) oriented gratings which are shown as red points in Fig. 2(a–c).

Using the concept of rotational invariance, we postulate that a scattering orientation $q\in {{\mathbb{S}}}^{2}$ can be recovered for a three-dimensional volume by measuring it from *N* poses *x*_*i*_, *i* = 1, 2, …, *N*, such that *s*(*x*_*i*_) = *q* and the vectors *t*(*x*_*i*_) consist of *N* points equally spaced on a circular trajectory. It can be seen that this condition is satisfied for *q* = [0, 1, 0]^*T*^ in Fig. 2(a). On the other hand, each of the 11 points of scheme *A*(0, 0, 11) measure a separate sensitivity vector when gratings are placed vertically (Fig. 2(b)) or diagonally (Fig. 2(c)). However, we can obtain the same blue and red points as Fig. 2(a) for vertical and diagonal gratings with the schemes *A*(0, 90, 11) and *A*(0, 45, 11), respectively. More generally, we can obtain an acquisition scheme *Y*(*q*, *S*, *N*) := {*y*_*i*_(*q*, *S*) := (*ψ*, *θ*, *ϕ*), *i* = 1, …, *N*} that fully measures the orientation *q* for the setup sensitivity *S*. In other words, *s*(*y*_*i*_(*q*, *S*)) = *q* for all *i*. To obtain a scheme *Y*(*q*, *S*, *N*), we transform *x*_*i*_  ∈ *A*(0, 0, *N*) to *y*_*i*_(*q*, *S*). Note from Fig. 2(a) that *A*(0, 0, *N*) = *Y*([0, 1, 0]^*T*^, [0, 1, 0]^*T*^, *N*). The method to calculate *Y*(*q*, *S*, *N*) from *A*(0, 0, *N*) is given in Algorithm 1.

Figure 2(d) shows an orientation vector ± *q* in red and the vectors ± *t*(*y*) in blue for all *y* ∈ *Y*(*q*, [0.7071, −0.7071, 0]^*T*^, 10). Using the procedure described above, we can design an acquisition scheme *Z*(*S*, *N*) that measures *L* scattering orientations fully and comprises of L*N poses for the given setup sensitivity *S*:

3 $$Z(S,N)=\{Y({q}_{l},S,N);l=1,\ldots ,L\}.$$

**Algorithm 1**: Calculate *Y*(*q*, *S*, *N*) from *A*(0, 0, *N*)

For every *x* ∈ *A*(0, 0, *N*)

1. Calculate Euler rotation matrix *R*(*x*).
2. Calculate *R*_*intermediate*_(*x*, *q*) such that *R*_*intermediate*_(*x*, *q*) · [0, 1, 0]*T* = *q*:
  4 $${R}_{intermediate}(x,q)=R(x)\cdot M([0,1,0{]}^{T},q),$$
  where $M({v}_{1},{v}_{2})\in {{\mathbb{R}}}^{3\times 3}$ is a matrix such that *v*_2_ = *M*(*v*_1_, *v*_2_) · *v*_1_ for all ${v}_{1},{v}_{2}\in {{\mathbb{S}}}^{2}$.
3. Estimate the orientation $u(x,q,S)\in {{\mathbb{S}}}^{2}$ measured by the rotation matrix *R*_*intermediate*_(*x*, *q*) given the setup sensitivity *S*:
  5 $$u(x,q,S)={R}_{intermediate}(x,q)\cdot S\mathrm{.}$$
4. Rotate *u*(*x*, *q*, *S*) to *q*:
  6 $${R}_{final}(x,q,S)={R}_{intermediate}(x,q)\cdot M(u(x,q,S),q\mathrm{).}$$
5. Compute the pose *y*(*q*, *S*) = (*ψ*, *θ*, *ϕ*) from the matrix *R*_*final*_(*x*, *q*, *S*) such that the absolute value of *ψ* in *y*(*q*, *S*) is minimized.

### Anisotropic X-ray Dark-Field Tomography

The goal of tomographic reconstruction of the anisotropic dark-field signal is to reconstruct the scattering information at each location within the measured object. In order to do so, we use the recently introduced AXDT method^18^. Here the scattering profile in each location of the object is modeled as a spherical function such that the process of reconstruction aims at recovering this field of spherical functions $\eta :{{\mathbb{S}}}^{2}\times {{\mathbb{R}}}^{3}\to {\mathbb{R}}$. In order to model the sensitivity vector *s*(*x*) and tomographic vector *t*(*x*) for every pose *x*, a weighting function $h:{{\mathbb{S}}}^{2}\times {{\mathbb{S}}}^{2}\times {{\mathbb{S}}}^{2}\to {\mathbb{R}}$ is used. Further, both the field of scattering functions as well as this weighting function are expressed in terms of real-valued spherical harmonics with their coefficients denoted as ${\eta }_{k}^{m}$ and ${h}_{k}^{m}$ respectively, where *k* specifies the degree and *m* the order of the spherical harmonics. With this notation and a truncation degree *K*, the dark-field measurement at the acquisition pose *x* can be approximately modelled by:

7 $$d(x)\approx \exp (-\frac{1}{4\pi }\sum _{k=0}^{K}\sum _{m=-k}^{k}{h}_{k}^{m}(s(x),t(x)){\int }_{T(x)}{n}_{k}^{m}(r)dr)\mathrm{.}$$

By using one of the standard discretization methods for line integrals of *p* = (−ln(*d*(*x*))) over the rays *T*(*x*) yielding a system matrix *P* and by forming weighting matrices ${W}_{k}^{m}$ according to ${h}_{k}^{m}(s(x),t(x))$, the reconstruction of the spherical harmonics coefficients of the field of scattering profiles reduces to solving the following linear equation system:

8 $$p=\sum _{k=0}^{K}\sum _{m=-k}^{m=k}{W}_{k}^{m}P{\eta }_{k}^{m}=\mathop{\underbrace{(\begin{array}{ccccc}{W}_{0}^{0}P & \cdots  & {W}_{K}^{-K}P & \cdots  & {W}_{K}^{K}P\end{array})}}\limits_{\,=A\,}(\begin{array}{c}{\eta }_{0}^{0}\\ \vdots \\ {\eta }_{K}^{-K}\\ \vdots \\ {\eta }_{K}^{K}\end{array}),$$

where we call *A* as the full system matrix. We solve this system using the conjugate gradient method with *K* = 4. Spherical harmonics of degree 4 are sufficient to describe the spherical function completely with the weighting function that is used.

### Null space estimation

One way to analyze the acquisition schemes and their effect on the reconstruction is to analyze the nature of the full system matrix *A*. An important aspect of a linear operator, such as *A* in the AXDT model described above, is the null space (or kernel) of *A*. It is defined as ker(*A*) := {*v*|*Av* = 0} and is of special interest as for *w* ∉ ker(*A*) and any *v* ∈ ker(*A*), the measurement does not change under addition i.e.,

9 $$Aw=A(w+v\mathrm{).}$$

The kernel provides a tool to analyze the matrix *A* and gives information about the uncertainty of a computed reconstruction. While it is well known that incomplete data leads to a larger nullspace, it is of special interest how elements of this space look like as they provide a relative insight of which regions are likely to be affected more/less.

Standard methods such as singular value decomposition (SVD) are typically used for computing the null space of such matrices. However, these methods rely on the full representation of the matrix. It should be noted that the system matrix *P* of the standard tomographic problem is typically too large to store in the memory of currently available computing devices. The full system matrix *A* for AXDT is larger by an additional factor of 2^*K*^ − 1 due to the weighting function. Therefore, we reconstruct one vector spanning a subspace of the nullspace of *A* by iteratively solving *Av* = 0 for *v*. Different vectors *v* ∈ ker(*A*) can be computed by starting from different initial guesses for *v*. We compute one component of the null space by starting with an initial guess of *v* such that:

10 $$v=\{{\eta }_{k}^{m};{\eta }_{k}^{m}=0\,{\rm{for}}\,k\ne 0,{\eta }_{k}^{m}=(\begin{array}{c}0.01\\ \vdots \\ 0.01\end{array})\,{\rm{for}}\,k=0\},$$

for all voxels. Since spherical harmonics are equivalent to a fourier series in terms of angular frequency, this initial guess is equivalent to starting with a uniform spherical function which is a good initial guess for clearly visualizing the effect of AXDT acquisition schemes. For computing the null space, we set the reconstruction volume size to 50 × 50 × 50, the detector size to 100 × 100 to limit computation times and use a parallel geometry assumption in order to eliminate errors at the edges due to forward and back-projection.

## Results

In this section, we present an analysis of the acquisition schemes designed in the previous section with respect to the setup geometry and its limitations. In addition, we use null space analysis to demonstrate the correlation of these schemes with the AXDT reconstruction. In the end, we corroborate the results presented in this section with experimental observations for an industrially relevant sample consisting of micrometer sized structures.

### Acquisition Schemes and Setup Geometry

**Figure 3:**
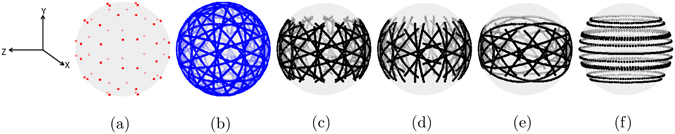
(**a**) A t-design with 56 uniformly distributed points. We aim to design trajectories that fully measure all of these scattering orientations. (**b**) Acquisition scheme *Z*([0.7071, −0.7071, 0]^*T*^, 100) with 2800 poses. Acquisition schemes (**c**) *Z*_*D*_(100) with 1676 poses, (**d**) *Z*_*H*_(100) with 1256 poses, (**e**) *Z*_*V*_(100) with 1284 poses, which measure all the points in (**a**) within the practical limitations of the setup with diagonally, horizontally and vertically aligned grating bars respectively. (**f**) acquisition scheme *W*(100).

In the previous section, we presented a method to design an acquisition scheme *Z*(*S*, *N*) that fully measures several sensitivity orientations on the unit sphere. The first step for designing such a scheme is to choose a set of orientations that we wish to measure. In order to reconstruct the spherical function using AXDT, it is required to choose orientations that are uniformly distributed on the unit sphere. One example of such sets of orientations are the t-designs presented by Hardin and Sloane^26^. We use these designs as they are a good choice for selecting uniformly distributed points on the sphere. However, any uniform distribution of points on the sphere can be used. We begin with a symmetric t-design consisting of 56 directions spread over the unit sphere as shown in Fig. 3(a). Since the AXDT model is symmetric around the origin, we use only 28 directions of this t-design spread over one half of the unit sphere.

Next, we generate an acquisition scheme:

11 $$Z({[0.7071,-0.7071,0]}^{T},100)=\{Y({q}_{i},{[0.7071,-0.7071,0]}^{T},100),\,i=1,\ldots ,28\},$$

consisting of 2800 poses. The vectors ± *t*(*x*) for all *x* ∈ *Z*([0.7071, −0.7071, 0]^*T*^, 100) are shown in Fig. 3(b). This acquisition scheme fully measures the 56 points shown in Fig. 3(a). However, it can be seen in Fig. 1 that the Eulerian cradle intercepts the beam for high values of *ψ*, hence, the setup is limited to −40 ≤ *ψ* ≤ 40. Therefore, all of the 2800 poses for the scheme *Z*(*S*, 100) cannot be measured. Figure 3(c) shows the points of Fig. 3(b) that can be measured with the condition |*ψ*| ≤ 40°.

**Figure 4:**
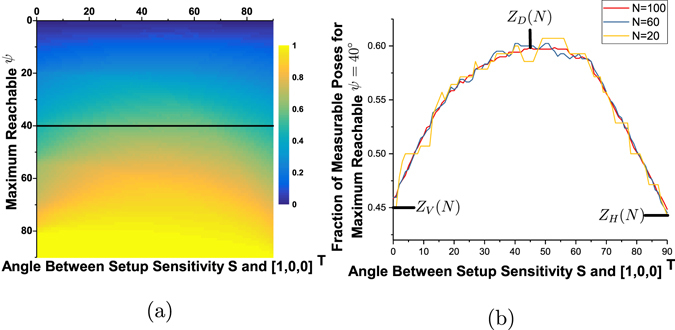
(**a**) 2D plot of the fraction of poses that can be measured with different grating arrangements and different *ψ* angle limitation. (**b**) Fraction of poses that can be measured with maximum reachable *ψ* = 40° for acquisition schemes *Z*(*S*, *N*). Red curve corresponds to the black line marked in (**a**). We study three points on these curves corresponding to vertical, diagonal and horizontal grating alignment denoted by *Z*_*V*_(*N*), *Z*_*D*_(*N*) and *Z*_*H*_(*N*) respectively.

The ratio of the points that can be measured (Fig. 3(c)) to the total number of desired points (Fig. 3(b)) is a function of the maximum reachable value of *ψ* and the setup sensitivity. Therefore, to study this effect, we calculate *Z*(*S*, *N*) for 91 values of *S* such that:

12 $$\langle S,[1,0,0]\rangle =\,\cos (\alpha );\alpha \in [{0}^{\circ },{1}^{\circ },{2}^{\circ },\ldots ,{90}^{\circ }],$$

where $\langle \cdot ,\cdot \rangle$ denotes the inner product. Figure 4(a) shows a 2D plot of the relative fraction of measurable poses (out of 2800) for the acquisition scheme *Z*(*S*, 100) for different values of reachable *ψ* ∈ [0°, 1°, 2°, …, 90°]. A line of this 2D plot for the maximum reachable *ψ* limit of 40° is shown in Fig. 4(b) (red curve). The point of maximum of this line plot corresponds to *Z*([0.7071, −0.7071, 0]^*T*^, 100), that is, when the grating bars are placed diagonally. In addition, similar line plots for acquisition schemes *Z*(*S*, *N*), *N* = 60, 20 are shown. We observe that the line plots for lower values of *N* have step-like artifacts arising from round-off errors which can be circumvented by using large number of sampling orientations. As it is difficult to actually align gratings at precise angles in most of the currently available setups, we only study the three extreme points marked in Fig. 4(b). We define the notation *Z*_*D*_(*N*), *Z*_*H*_(*N*) and *Z*_*V*_(*N*) to denote acquisition schemes with diagonal, horizontal and vertical arrangement of grating bars respectively. Figure 3(c–e) shows the vectors ± *t*(*x*) for the measurable poses of acquisition schemes *Z*_*D*_(100), *Z*_*H*_(100), and *Z*_*V*_(100), respectively, assuming that poses with |*ψ*| > 40° cannot be measured. It is evident from these results that the maximum amount of poses can be probed by placing the gratings such that the grating lines are aligned diagonally. From now on, we will assume that all acquisition schemes *Z*(*S*, *N*) are truncated at |*ψ*| = 40°, since this is the practical limit of our setup.

### Correlation of the Design of Acquisition Schemes with AXDT Reconstruction

We presented a method to design acquisition schemes by extending the concept of circular acquisition trajectories normally used in conventional CT to the special case of XTT/AXDT. In this section, we visualize the null space of the new schemes to demonstrate the correlation between the acquisition schemes and the AXDT reconstruction.

It was outlined above that the acquisition trajectories are truncated due to the physical limitations of the setup. This means that we cannot measure the full tomographic trajectory *Y*(*q*_*i*_, *S*, *N*) for all the orientations *q*_*i*_, *i* = 1, …, 28. The missing angles lead to circular trajectories with missing wedges similar to the ones in limited angle tomography. In standard tomography, the null space is easily understood in terms of the Fourier Slice theorem. However, the Fourier Slice theorem does not carry over to our system due to its sensitivity specificity. Hence, it is not possible to assume that similar limited angle artifacts can also be seen for the case of AXDT. Moreover, all of the poses for *Z*(*S*, *N*) are used for the reconstruction of the spherical harmonic coefficients in AXDT reconstruction, and there is no direct reconstruction of individual components corresponding to each of the truncated trajectory. However, we postulate that our acquisition schemes correlate well to the reconstruction process of AXDT and, hence, some effect of the limited angle trajectories should be visible in the corresponding spherical function.

**Figure 5:**
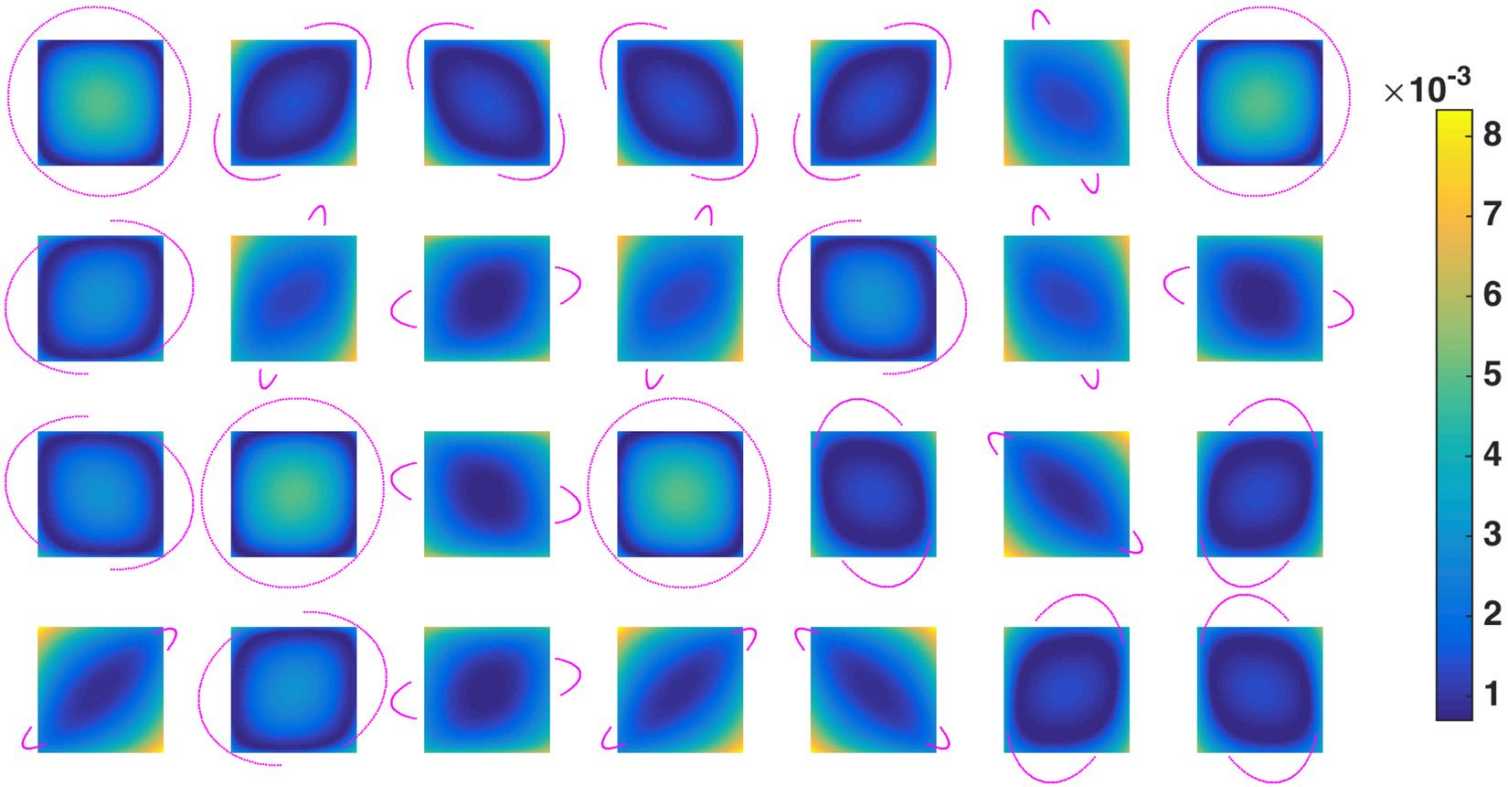
Null space components for each of the 28 points in Fig. 3(a) calculated using the acquisition scheme *Z*_*D*_(100) (Fig. 3(c)). The magenta points show the trajectories for each component truncated for maximum reachable |*ψ*| = 40° and the correponding images show the null space averaged over all *x* − *z* planes.

To check this hypothesis, we estimate the null space for the proposed schemes as explained previously. Next, we probe the reconstructed null space (in terms of spherical coefficients) at the specific points *q*_*i*_, *i* = 1, …, 28, in order to evaluate the effect of the missing wedges for these individual scattering orientations. Figure 5 shows the null space components for the scheme *Z*_*D*_(100) (similar figures for *Z*_*V*_(100) and *Z*_*H*_(100) are provided in the Supplementary Material). The magenta points show the tomographic trajectory for all components *q*_*i*_ as projected onto the *x* − *z* plane. Analogously, the images show the null space averaged over all *x* − *z* planes for the corresponding component after the first iteration. The null space artifacts caused by the *ψ* truncation can be seen explicitly in the individual components reconstructed with AXDT.

### Comparison to Schemes Presented by Malecki *et al*

In this section, we compare the schemes in Fig. 3(c–e) to the ones previously used by our group^16–18, 21^. Such a scheme can be written as:

13 $$\begin{array}{rcl}W(N) & = & \{w=(\psi ,\theta ,\varphi );\,\psi \in \{{0}^{\circ },{20}^{\circ },{40}^{\circ }\},\theta \in \{{0}^{\circ },{30}^{\circ },{60}^{\circ },{90}^{\circ }\},\\ \quad \,\,\,\,\varphi  & \in  & \{{0}^{\circ },\frac{{360}^{\circ }}{N},\ldots ,{360}^{\circ }-\frac{{360}^{\circ }}{N}\}\}.\end{array}$$

The trajectory ± *t*(*w*) for all *w* ∈ *W*(100) is shown in Fig. 3(f). Here we assume vertical grating alignment which is the most commonly used configuration.

**Figure 6:**
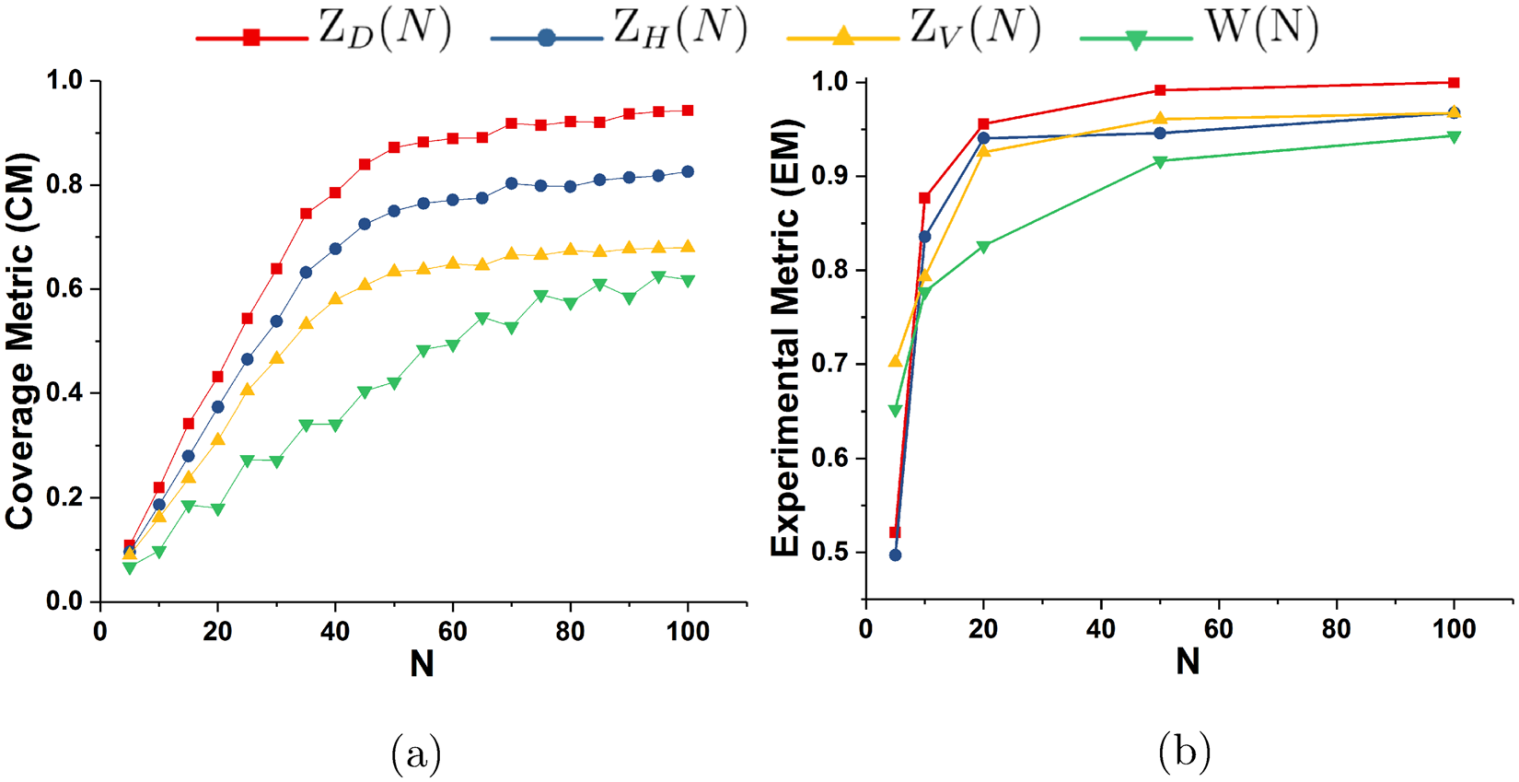
(**a**) Coverage Metric (*CM*) and (**b**) Experimental Metric (*EM*) as a function of *N* for four different acquisition schemes.

In order to compare the proposed schemes for different grating orientations and *W*(*N*), we use the Coverage Metric (*CM*) proposed in our previous work^22^. This is a metric which determines the degree up to which an acquisition scheme measures all orientations on the unit sphere or, in other words, *CM*(*X*) provides a measure of the efficiency of any acquisition scheme *X*. The usability of *CM* as a valid metric for XTT acquisition schemes was established in ref. 22. It can be directly applied to AXDT as well, since the two methods only differ in the reconstruction method and the acquisition protocol is identical. Figure 6(a) shows the coverage metric for *Z*_*D*_(*N*), *Z*_*H*_(*N*), *Z*_*V*_(*N*), and *W*(*N*) for different values of *N*.

## Experimental Results

**Figure 7:**
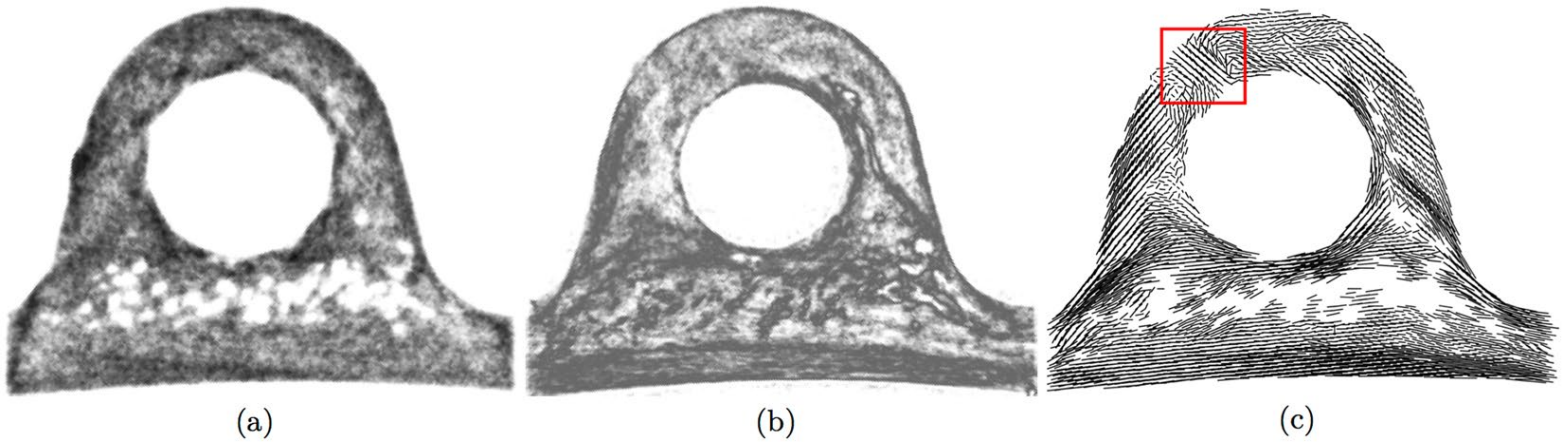
A slice of (**a**) the conventional CT volume, (**b**) the isotropic scattering component, and (**c**) the 3D vectors reconstructed using AXDT for a thermoplastic short fibre moulding sample measured at a spatial resolution of approximately 80 μm. The red box marks a feature that cannot be seen in either of (**a**) or (**b**).

In this section, we present experimental results to support the observations made in the previous sections. We measured a circular thermoplastic short fibre moulding part, composed of fibres that are 7 μm thick and 200 μm long, at a resolution of approximately 80 μm. We employed 7 phase steps with 1 sec. exposure per step using a setup^7^ comprising of an X-ray WorX micro-focus X-ray tube (operated at voltage 60 kVp and power 25 W) and a Varian PaxScan 2520DX detector (pixel size 127 μm). The three gratings with periods of 10 μm, 5 μm and 10 μm, respectively, were arranged in the first fractional Talbot configuration at a design energy of 45 keV. Since we obtain both the dark-field and attenuation signal in a grating interferometer, we can also reconstruct the attenuation volume. One slice of the attenuation data is shown in Fig. 7(a). Obviously, the imaging resolution is not sufficient to resolve the fibres with a diameter of 7 μm. However, we can resolve the porosity as seen in the bottom of Fig. 7(a).

Next, we perform AXDT reconstruction. A slice of ${\eta }_{0}^{0}$, which is the isotropic component of the dark-field signal, is shown Fig. 7(b). Figure 7(c) shows the main orientation in every third voxel as extracted from the spherical function. The vectors have been masked with the attenuation signal to avoid the undesired effect of edge-scattering at the pores^27, 28^. The red box in Fig. 7(c) shows a feature in this sample which cannot be seen in either of 7(a) or 7(b). This is a weld-line and is only revealed by extracting the orientations of the fibres in the region using AXDT. Very high resolution micro CT may be required to directly resolve this structure but then a sample of this dimension (28 × 23 × 21 mm^3^) cannot be measured at once and one would have to resort to multiple tomographies or even to destroying the sample.

**Figure 8:**
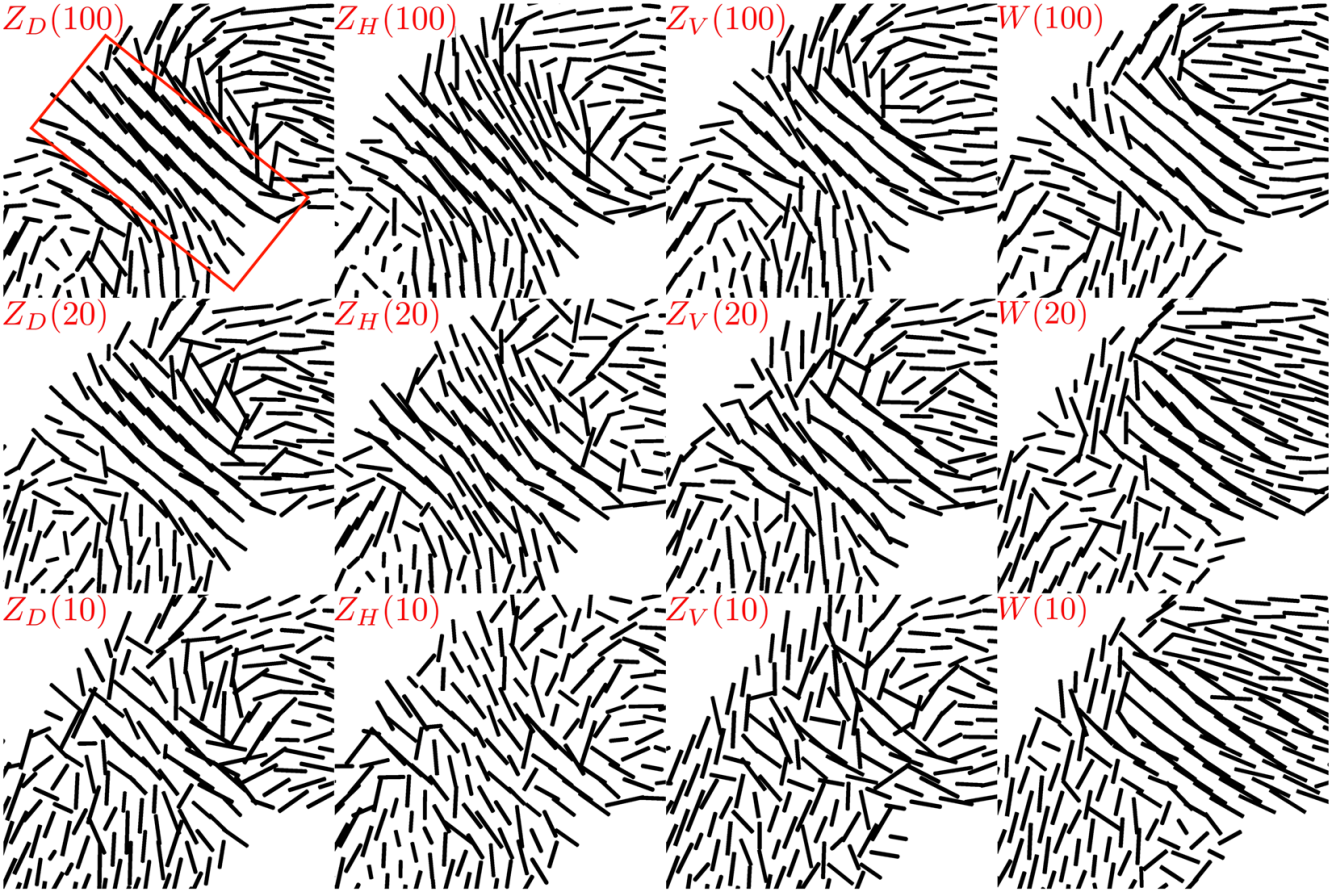
Structure orientations calculated using AXDT in the region-of-interest (indicated by the red box in Fig. 7(c)) for different acquisition schemes. Each column corresponds to acquisition schemes and every row shows the four schemes with the same value of *N*. The red box indicates the region-of-interest used for calculating the Experimental Metric.

In order to evaluate the effect of the grating orientation on the reconstruction result, we show the region marked in Fig. 7(c) for different acquisition schemes in Fig. 8. The columns in Fig. 8 correspond to three different values of *N* = {100, 20, 10} for a specific scheme, while the rows show four different schemes for the same value of *N*. Next, we define an Experimental Metric, *EM*(*X*), for an acquisition schemes *X* as:

14 $$EM(X)=\frac{1}{I}\sum _{i=1}^{I}|\langle {U}_{i}(X),{U}_{i}({Z}_{D}(100))\rangle |$$

where $\langle \cdot ,\cdot \rangle$ is the standard scalar product, *U*_*i*_(*Z*_*D*_(100)) and *U*_*i*_(*X*) denote the structure orientation for the voxel index *i* = 1, …, *I* in the region-of-interest (red rectangle in Fig. 8) calculated using the acquisition scheme *Z*_*D*_(100) and an arbitrary scheme *X*, respectively. The value of the Experimental Metric for different schemes is shown in Fig. 6(b).

## Discussion

We present a new technique to design acquisition schemes for directional dark-field tomographic imaging. We use the concept of rotational invariance to design tomographic trajectories that fully measure a unique component of the three dimensional scattering function. However, we show that all of the desired poses cannot be measured in a regular setup. In fact, this is a problem for most of the setup configurations, since it is always difficult to measure along the axis of the sample mount with X-rays. Therefore, we show the fraction of poses that can be measured for different setup configurations in Fig. 4(a,b). We observe that while it is possible to measure all the information with any grating orientation in an ideal setup, the grating orientation starts to play a major role when the setup limitation, that is the availability of the angle *ψ* only up to a certain value, is enforced. It can be seen that we can optimize the amount of measurable poses by placing the gratings diagonally.

Next, we study the effect of the aforementioned setup limitation by visualizing the null space of the AXDT operator. It can be seen in Fig. 5 that the *ψ* limitation leads to missing wedges (magenta points) in the tomographic trajectories for most of the orientations. We note that the effect of these missing wedges can be directly seen in the null space of the corresponding component. This is an interesting finding because of the fact that even though the AXDT operator uses all of the poses at the same time to reconstruct the spherical function, we are still able to see the effect of the limited angle trajectories at the corresponding sensitivity points on the reconstructed field. More importantly, the null space visualization is proof that the initial concept of calculating tomographic trajectories which provide invariant dark-field signal for certain pre-defined orientation vectors works exactly as we expect it to. Please note that the fact that we only get one component of ker(*A*) is not a limitation for our work since we compute this component in the exact same way for all the orientations.

We use the Coverage Metric (*CM*)^22^ to compare the new schemes *Z*_*D*_(*N*), *Z*_*H*_(*N*), *Z*_*V*_(*N*) among themselves and to the schemes that have been used by our group in previous works *W*(*N*). It can be seen in Fig. 6(a) that the value of *CM* decreases with the value of *N* for all four schemes and that the new schemes outperform the previously used scheme *W*(*N*). Moreover, *Z*_*D*_(*N*) has the highest value of *CM* amongst the new schemes. This implies that diagonal grating alignment is the most efficient of the proposed schemes (as also seen in Fig. 4), followed by horizontal alignment, and vertical grating alignment is the least favorable.

Finally, we show an example of the application of AXDT to an industrially relevant composite material. We show in Fig. 7(c) that AXDT is able to resolve orientations of fibres with sizes that are much below the resolution of the imaging system. Moreover, the fibre orientations calculated using AXDT reveal a particular feature (weld-line) in the sample which cannot be seen in conventional attenuation or even the isotropic component of the dark-field signal. We compare, qualitatively in Fig. 8 and quantitatively in Fig. 6(b), the new schemes *Z*(*S*, *N*) for three different grating orientations and the old schemes *W*(*N*). We can see in Fig. 8 that although the weld-line is clearly visible in all the schemes with *N* = 100 (first row), the scheme *Z*_*D*_(100) provides the most comprehensible distinction of the weld-line. More importantly, the quality of this result is maintained for *Z*_*D*_(20) and *Z*_*H*_(20), while significant deterioration can be seen for *Z*_*V*_(20) and *W*(20). The vectors reconstructed with the schemes *W*(*N*) (last column in Fig. 8) seem to be oriented in certain preferred directions and the variations are lost. This is due to the fact that the schemes *W*(*N*) provide an uneven sampling of the unit sphere^22^ and the reconstruction is biased towards a partial reconstruction of the most commonly sampled scattering orientations.

In Fig. 6(b), we compare the performance of the schemes with respect to the scheme *Z*_*D*_(100), which is assumed as the reference dataset. Here, we can see that the vectors in the region-of-interest deviate most from the reference for the schemes *W*(*N*). Also, we observe that the trend of the graph for the four schemes matches the corresponding trend observed in Fig. 6(a). This observation supports our claim that the new type of acquisition schemes provide better results than the old ones. Moreover, we can also conclude that diagonal grating orientation is the most favorable followed by horizontal and vertical alignment of gratings for the new schemes. It should also be noted that *Z*_*D*_(20) corresponds to a measurement time of only ~2 hours and is still of comparable quality to *Z*_*D*_(100) which requires ~10 hours of measurement. This reduction in acquisition time is a substantial improvement compared to the long measuring times of ~10 hours required for the schemes used previously^16, 17, 22^.

## Conclusion

In this work, we present a more efficient method to design acquisition schemes for tomographic imaging of the directional dark-field signal and present results of diagonal grating orientation being the most optimal setup configuration for these schemes. Finally, we also show that the new schemes with diagonal grating alignment allow us to obtain good image quality with only ~2 hours of measurement time instead of the ~10 hours of measurements that was used previously.

## Electronic supplementary material

Supplementary Information
