# Supplementary material for: Design of Acquisition Schemes and Setup Geometry for Anisotropic X-ray Dark-Field Tomography (AXDT)
Source: Sci Rep. 2017 Jun 9;7:3195. doi: 10.1038/s41598-017-03329-0 (PMC5466688; doi:10.1038/s41598-017-03329-0)
Supplement: Supplementary file 1 — Supplementary Information [file 41598_2017_3329_MOESM1_ESM.pdf]

Supplementary Information: Design of Acquisition Schemes and Setup Geometry for Anisotropic X-ray Dark-Field Tomography (AXDT)

Y. Sharma[1,2*], F. Schaff[1], M. Wieczorek[2], F. Pfeiffer[1,3,4], and T. Lasser[2]

[1]Chair of Biomedical Physics, Department of Physics and School of BioEngineering, Technical University of Munich, 85748 Garching, Germany
[2]Computer Aided Medical Procedures, Technical University of Munich, 85748 Garching, Germany
[3]Department of Diagnostic and Interventional Radiology, Klinikum rechts der Isar, Technical University of Munich, 81675 München, Germany
[4]Institute for Advanced Study, Technical University of Munich, 85748 Garching, Germany
[*]y.sharma@tum.de

ABSTRACT

Figure 5 in the manuscript shows the null space components for the acquisition scheme $Z_D(100)$. Here, we provide the corresponding figures for the acquisition schemes $Z_H(100)$ and $Z_V(100)$.

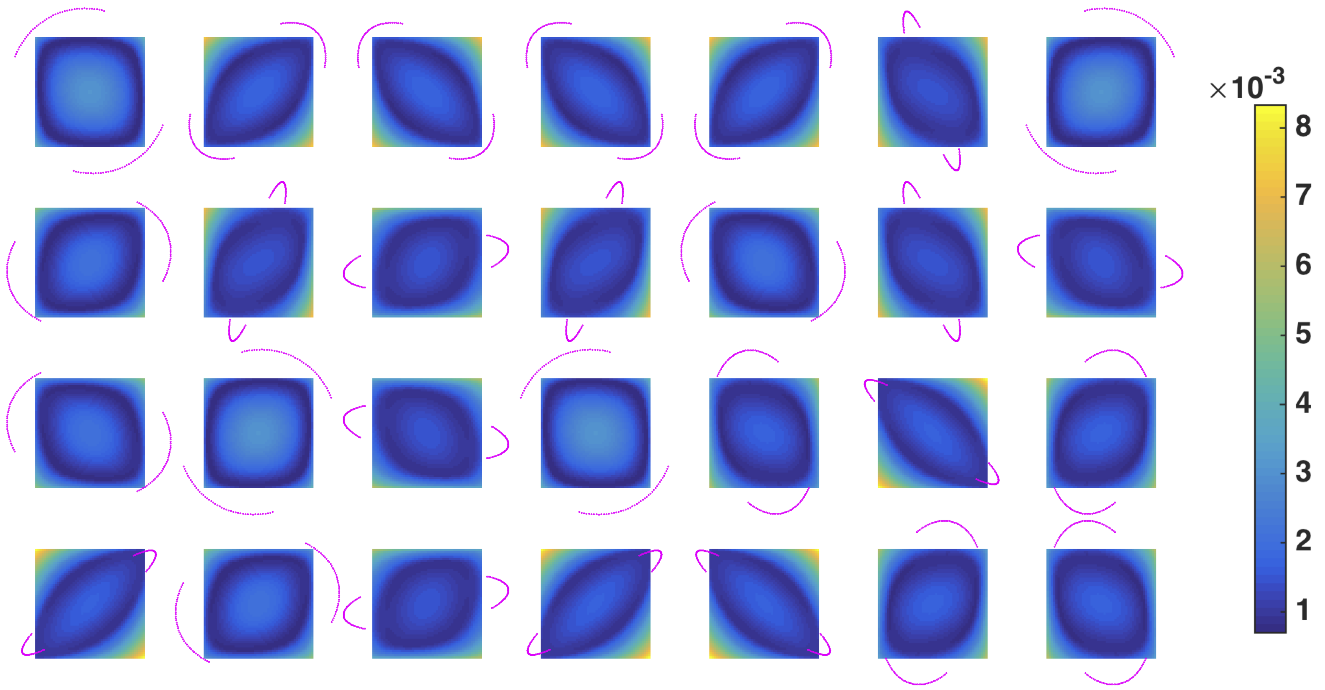

Figure S1. Null space components for each of the 28 points in Figure 3(a) in the manuscript calculated using the acquisition scheme $Z_H(100)$ (Figure 3(d) in the manuscript). The magenta points show the trajectories for each component truncated for maximum reachable $|\psi| = 40°$ and the correponding images show the null space averaged over all $x - z$ planes.

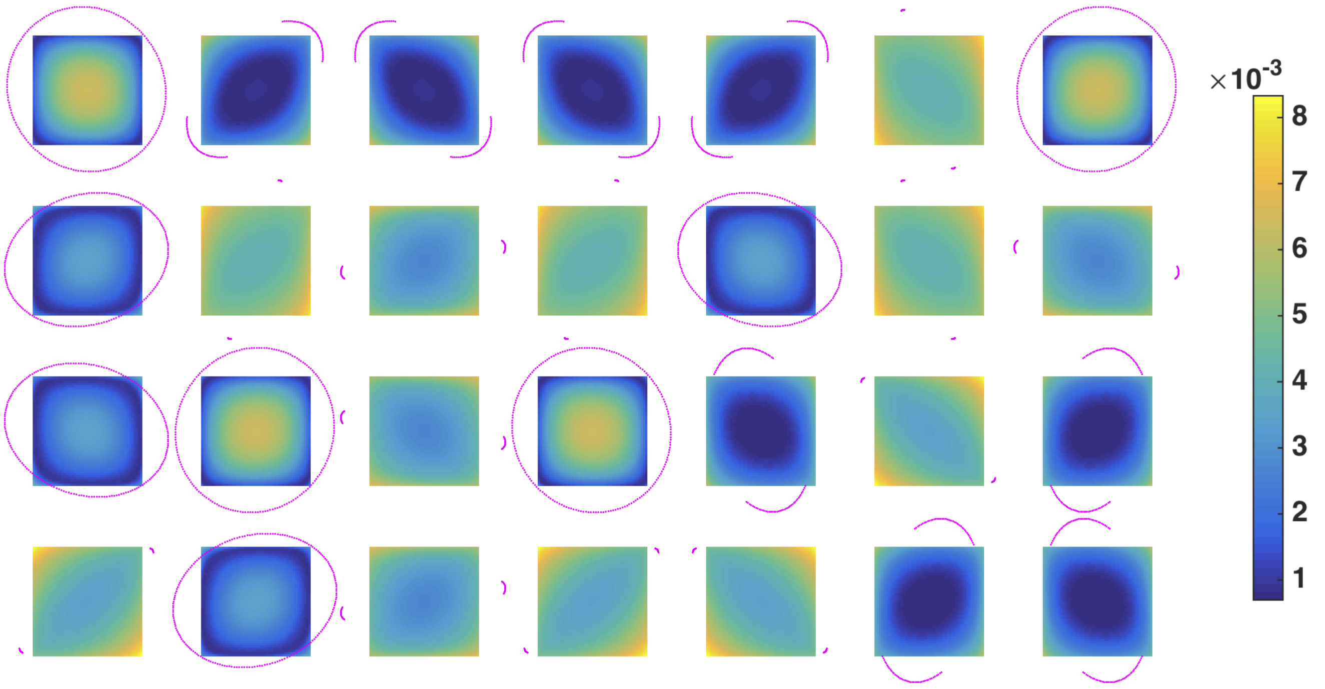

Figure S2. Null space components for each of the 28 points in Figure 3(a) in the manuscript calculated using the acquisition scheme $Z_V(100)$ (Figure 3(e) in the manuscript). The magenta points show the trajectories for each component truncated for maximum reachable $|\psi| = 40°$ and the correponding images show the null space averaged over all $x - z$ planes.
